# Reduced alcohol preference and intake after fecal transplant in patients with alcohol use disorder is transmissible to germ-free mice

**DOI:** 10.1038/s41467-022-34054-6

**Published:** 2022-10-19

**Authors:** Jennifer T. Wolstenholme, Justin M. Saunders, Maren Smith, Jason D. Kang, Phillip B. Hylemon, Javier González-Maeso, Andrew Fagan, Derrick Zhao, Masoumeh Sikaroodi, Jeremy Herzog, Amirhossein Shamsaddini, Marcela Peña-Rodríguez, Lianyong Su, Yun-Ling Tai, Jing Zheng, Po-Cheng Cheng, R. Balfour Sartor, Patrick M. Gillevet, Huiping Zhou, Jasmohan S. Bajaj

**Affiliations:** 1 VCU-Alcohol Research Center and Department of Pharmacology and Toxicology, Virginia Commonwealth University, Richmond, VA USA; 2 Department of Physiology and Biophysics, Virginia Commonwealth University, Richmond, VA USA; 3 Microbiology and Immunology, Virginia Commonwealth University, Richmond, VA USA; 4 Division of Gastroenterology, Hepatology and Nutrition, Virginia Commonwealth University and Richmond VA Medical Center, Richmond, VA USA; 5 Microbiome Analysis Center, George Mason University, Manassas, VA USA; 6 National Gnotobiotic Rodent Research Center, Center for Gastrointestinal Biology and Disease, University of North Carolina at Chapel Hill, Chapel Hill, NC USA; 7 University Center for Health Sciences, University of Guadalajara, Guadalajara, Jalisco Mexico; 8 Department of Medicine, Division of Gastroenterology and Hepatology, University of North Carolina at Chapel Hill, Chapel Hill, NC USA

**Keywords:** Alcoholic liver disease, Liver cirrhosis, Microbiota

## Abstract

Alcohol use disorder is a major cause of morbidity, which requires newer treatment approaches. We previously showed in a randomized clinical trial that alcohol craving and consumption reduces after fecal transplantation. Here, to determine if this could be transmitted through microbial transfer, germ-free male C57BL/6 mice received stool or sterile supernatants collected from the trial participants pre-/post-fecal transplant. We found that mice colonized with post-fecal transplant stool but not supernatants reduced ethanol acceptance, intake and preference versus pre-fecal transplant colonized mice. Microbial taxa that were higher in post-fecal transplant humans were also associated with lower murine alcohol intake and preference. A majority of the differentially expressed genes (immune response, inflammation, oxidative stress response, and epithelial cell proliferation) occurred in the intestine rather than the liver and prefrontal cortex. These findings suggest a potential for therapeutically targeting gut microbiota and the microbial-intestinal interface to alter gut-liver-brain axis and reduce alcohol consumption in humans.

## Abstract (web summary)

Gut microbiota composition is altered in patients with alcohol use disorder, and fecal microbiota transplant reduced alcohol craving in patients with alcohol use disorder and liver cirrhosis in a phase 1 clinical trial. Here the authors used stool samples collected in the trial to report that this phenotype is transmissible via microbial transfer to germ free mice, as assessed by reduced ethanol acceptance, intake and preference.

## Introduction

Alcohol use disorder (AUD) is a major cause of reduced life expectancy worldwide, and this misuse has increased exponentially during the pandemic^1,2^. Excessive alcohol consumption can lead to end-organ damage across several organs, but the liver and resultant cirrhosis is a significant complication^3^. Cirrhosis alone, regardless of AUD, is associated with microbial dysbiosis and defects in the intestinal mucosal barrier, which can worsen in the presence of alcohol misuse^4–6^. This situation is complicated by the lack of adequate study of AUD-related pharmacotherapy in this advanced population^3^. Therefore, in these patients, it becomes even more important to reduce alcohol misuse to prevent further liver injury.

Recent preclinical and clinical studies have demonstrated that fecal transplant of microbiota from patients with AUD can change the intestinal barrier and affect brain function^7–11^. Specific changes related to blood metabolome were noted to modulate these behaviors^12^. Moreover, in patients with AUD, there are specific changes in the gut microbiome and intestinal barrier that are unique to cirrhosis versus alcohol and selected taxa change after alcohol abstinence^10,13^. The next step is to determine if changing the microbiota could beneficially reduce the craving and alcohol intake in patients with AUD and the potential mechanisms behind these changes. We performed a placebo-controlled randomized clinical trial in men with cirrhosis and AUD in which fecal microbiota transplant (FMT) from a donor enriched in Lachnospiraceae and Ruminococcaceae led to short-term reduction in alcohol craving and consumption with a concomitant increase in short-chain fatty acids in the FMT group but not placebo^14^. This also led to an overall reduction in AUD-related adverse events over 6 months in the FMT group compared to placebo^14^. However, the role of microbial alterations on the gut-liver-brain axis needs to be investigated further. Our aim was to determine if alcohol craving improvement in humans after FMT could be transmitted to mice through microbial transplantation.

We had previously shown that fecal microbiota transplant reduced alcohol craving in humans with cirrhosis. Here we show that the reduction in craving and alcohol preference was transmissible to germ-free mice only when live bacteria and not germ-free supernatants were used for colonization. This differential colonization was associated with alterations in the gut immune-inflammatory response through short-chain fatty acids.

## Results

### Clinical trial showed reduction in alcohol intake and craving after FMT in humans

**Fig. 1:**
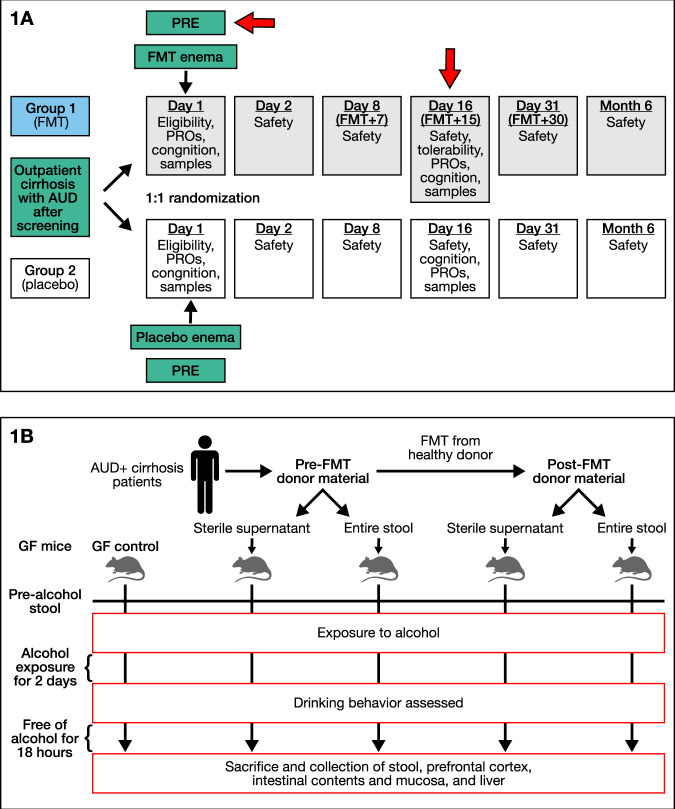
Study design. **A** Human RCT design with red arrows at the timepoints stools from humans were collected for colonization into germ-free mice. **B** Animal experiment design from human to male germ-free C57BL/6J mouse stool transfer and sterile supernatant collection with stool collection pre and post-alcohol exposure with necropsy 18 h post-last alcohol exposure. A group of germ-free mice were also used as controls. *N* = 6–14 mice/group.

As previously published, we included 20 men with AUD-cirrhosis [65 ± 6.4 years, MELD 8.9 ± 2.7] were included (Fig. 1A)^14^. Groups were comparable with respect to demographics, cirrhosis, and AUD severity at baseline. Liver function and safety labs were stable over time. Craving reduced significantly in 90% of FMT-assigned versus 30% in placebo (*p* = 0.02). This was accompanied by lower urinary ethylglucuronide (Etg) (*p* = 0.03). These were not seen in the placebo patients (Supplementary Table 1).

### Alcohol intake and preference reflected the clinical trial results and was not recapitulated with sterile supernatants

We included 28 male 10–15 week-old C57BL/6J GF mice obtained from the UNC National Gnotobiotic Rodent Resource Center, half of whom received the pooled pre-FMT stools by gavage on 3 consecutive days and the rest received pooled post-FMT stools (Fig. 1B) in the initial experiment. No obvious sickness behavior was noted, nor were body weights different between the groups.

In the subsequent experiment, we included 18 male 10–15 week-old GF C57BL/6J, six of whom remained GF without colonization, and six each were administered the pre-FMT and post-FMT supernatants by gavage respectively. Two of the post-FMT supernatant gavage mice were found to be sickly and on dissection were found to have gavage-related injury; they were excluded from the analysis.

Since the two experiments (Pre vs Post-FMT intact stool colonization) and supernatants (Pre vs Post-FMT vs GF controls) were done at separate times, we analyzed these separately.

**Fig. 2:**
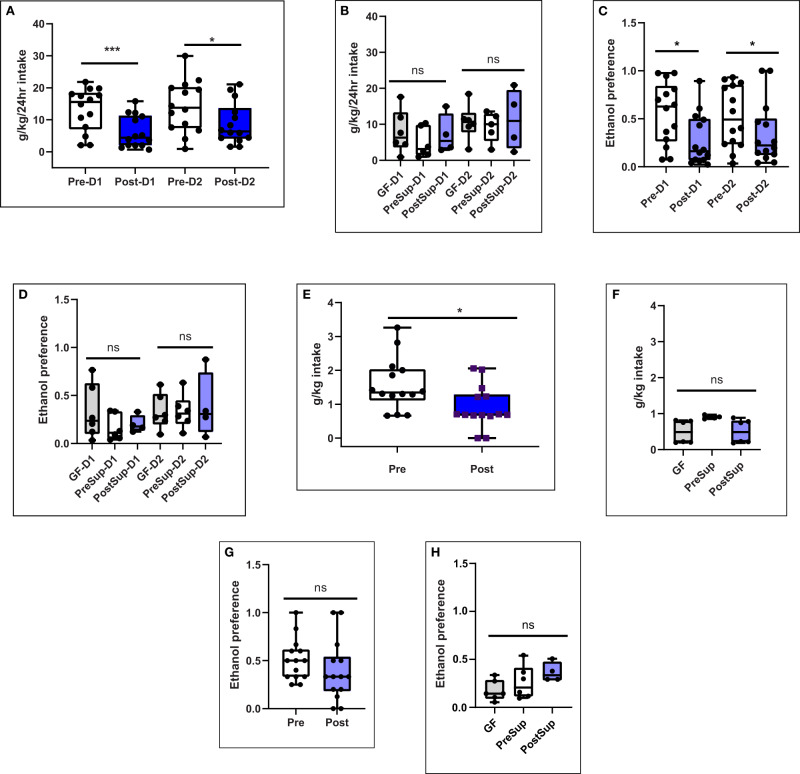
Alcohol Intake and preference is reduced in post-FMT male mice. **A**, **B** 24-hour two-bottle choice alcohol intake for 20% ethanol or water on days 1 and 2. **A** Pre and post-FMT stool colonized mice (D1 comparison *p* = 0.0058, D2 comparison *p* = 0.035) using one-sided Mann–Whitney tests, **B** Germ-free mice and those colonized using sterile supernatants from pre and post-FMT mice were not significantly different using using one-sided Kruskal–Wallis tests. **C** Pre and post-FMT stool colonized mice, **D** Germ-free mice and those colonized using sterile supernatants from pre and post-FMT mice. **E**–**H** Initial alcohol intake and preference measured during the 2-h initial binge on day 1. **E** Pre-FMT stool colonized mice had lower intake in the binge than post-FMT stool colonized mice (*p* = 0.03, Mann–Whitney test one-sided), **F** Germ-free mice and those colonized using sterile supernatants from pre and post-FMT mice were not significantly different on the binge, **G** Initial alcohol acceptance measured as a 2-h initial binge on day 1 was statistically similar across pre/post-FMT stool or H: between germ-free mice and those administered the supernatants.Pre Pre-FMT entire stool, Post Post-FMT entire stool, GF germ-free, PreSup GF supernatant Pre-FMT, PostSup GF supernatant Post-FMT, D1 day 1, D2 day 2. ***p < 0.001,*p < 0.05, NS not significant. Data presented as median, 95% CI (boxplot) with individual values and the entire range in the error bars. 14 mice per group for entire stool. 6 per group in GF, PreSup and 4 in PostSup experiments.

On day 16 post-colonization, mice underwent two days of two-bottle choice drinking for 20% (v/v) ethanol vs. water. Ethanol intake and preference during the two days of access were significantly reduced in post-FMT mice(Fig. 2A). The main effect of treatment (intake: *F*_1,26_ = 8.814, *p* = 0.006 and preference: *F*_1,26_ = 7.876, *p* = 0.009) was found by two-way repeated-measures ANOVA, which showed a significantly lower intake and preference for alcohol in those colonized with post-FMT compared to pre-FMT. Initial ethanol acceptance was measured as ethanol intake at two hours and was also significantly lower in post-FMT mice as compared to pre-FMT mice (*p* = *t*-test *p* = 0.009, Fig. 2E). There was also a lower preference for alcohol in post-FMT vs pre-FMT mice that were colonized with stool over days 1 and 2, and there was a trend towards significance during the binge (Fig. 2E, F). Total liquid consumed by the mice (i.e. 20% ethanol and tap water) or body weight was not different between the groups for any measure (Tables S2, 3).

There were no differences in alcohol intake or preference in the mice colonized by pre-FMT sterile supernatant compared to post-FMT and GF controls (Fig. 2C, D, G, H).

### Favorable changes in intestinal milieu and barrier function were seen with post-FMT stool colonization

**Fig. 3:**
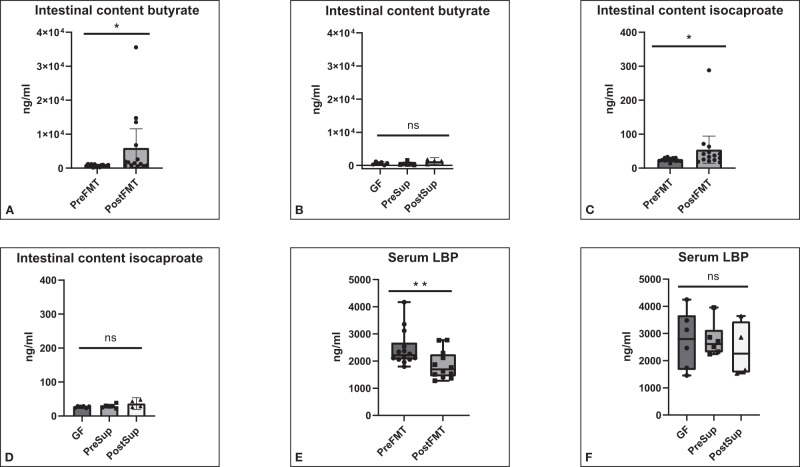
Intestinal contents SCFA (short-chain fatty acids) increased while serum LBP decreased with post-FMT stool colonization compared to pre-FMT. **A** Butyrate after colonization with entire stool, **B** Butyrate in germ-free mice and those colonized using sterile supernatants from pre and post-FMT mice. **C** Isocaproate after colonization with entire stool, **D** Isocaproate in germ-free mice and those colonized using sterile supernatants from pre and post-FMT mice. Data presented as mean and 95% CI (boxplot) with individual values and the entire range in the error bars. **E** Serum lipopolysaccharide binding protein (LBP) reduced after colonization with entire stool in post-FMT vs pre-FMT (*p* = 0.0074 Mann–Whitney test), **F** Serum LBP in germ-free mice and those colonized using sterile supernatants from pre and post-FMT mice (*p* = 0.88 Kruskal–Wallis test). Data presented as median and 95% CI (boxplot) and individual values and the entire range in the error bars. Pre Pre-FMT entire stool, Post Post-FMT entire stool, GF germ-free, PreSup GF supernatant Pre-FMT, PostSup GF supernatant Post-FMT, D1 day 1, D2 day 2. ***p < 0.001,*p < 0.05, NS not significant. 14 mice per group for entire stool. 6 per group in GF, PreSup and 4 in PostSup experiments.

There was a significant increase in butyrate and isocaproate levels in the intestinal contents after FMT compared to pre-FMT (Fig. 3A–D). No changes were seen in the GF and supernatant-treated mice. No other change in SCFAs were seen (Table 1). Serum LBP levels were significantly lower post-FMT vs pre-FMT but no changes were seen when GF and the two supernatant groups were compared (Fig. 3E, F).

**Table 1:** Intestinal content SCFA analysis

Ng/ml	Pre and post FMT intact stool			GF and sterile supernatant experiment			
	Pre FMT	Post FMT	*P* value	GF	Pre Sup	Post Sup	*P* value
Acetate	9630 (7730)	10,655 (19,629)	0.96	17,578 (20,810)	14,196 (14,507)	8553 (49,582)	0.65
Propionate	644 (770)	643 (3636)	0.43	797 (1458)	239 (1665)	308 (2298)	0.65
Butyrate	802 (485)	1357 (2660)	0.02	348 (798)	246 (822)	409 (1432)	0.60
Isobutyrate	59.2 (57.0)	60.6 (118.0)	0.73	18.7 (157.4)	56.2 (38.5)	45.2 (44.3)	0.33
Isocaproate	23.5 (8.5)	38.9 (18.1)	0.03	27.85 (5.53)	29.00 (10.75)	28.90 (18.65)	0.50

Data presented as median (IQR); Mann–Whitney and Kruskal–Wallis analysis were performed.

### Post-FMT mice had altered fecal microbiota which reflected changes seen in Post-FMT humans

**Fig. 4:**
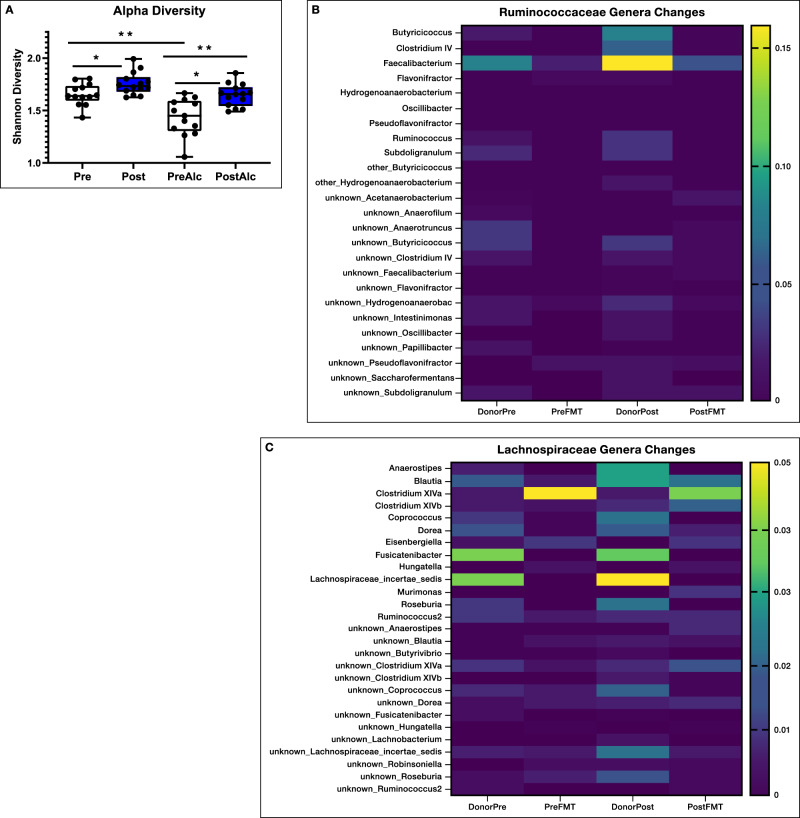
Stool microbial diversity and transmission of Lachnospiraceae and Ruminococcaceae constituents. **A** Shannon diversity index of stool microbiota showed lower alpha-diversity in pre-FMT versus post-FMT even before alcohol exposure. Alcohol exposure further worsened this in both groups. Pre Pre-FMT but not exposed to alcohol, Post Post-FMT but not exposed to alcohol, PreAlc Pre-FMT after alcohol exposure, PostAlc Post-FMT after alcohol exposure, **p < 0.01, *p < 0.05, Mann–Whitney for unpaired and Wilcoxon signed rank test for paired analyses (before/after alcohol) one-sided. There were 14 mice per group. Data presented as median, 95% CI (boxplot) with individual values and the entire range in the error bars. **B** Ruminococcaceae and **C** Lachnospiraceae genera heatmap of average relative abundance from human donors pre and post to GF mice. DonorPre: combined stools from patients with cirrhosis who were actively drinking that were collected before the FMT from a healthy human, DonorPost combined stools from the same patients with cirrhosis in DonorPre collected 15 days after the FMT from a healthy human, Pre-FMT stools from germ-free mice colonized with stools from DonorPre, Post-FMT stools from germ-free mice colonized with stools from DonorPost. Higher relative abundance of several genera belonging to Ruminococcaceae and Lachnospiraceae in DonorPost vs DonorPre, which was reflected in their respective mouse recipients.

The analyses shown in Fig. 4A show that diversity of stool microbiota was lower in pre-FMT versus post-FMT mice before and after alcohol drinking and the extent of this reduction after 48 h of ethanol access was higher in the pre-FMT mouse group. None of the GF mice or those that received GF supernatants had microbial growth as expected. Comparison of Lachnospiraceae and Ruminococcaceae genera showed good engraftment compared to pre- and post-FMT donors and recipients (Fig. 4B, C).

**Fig. 5:**
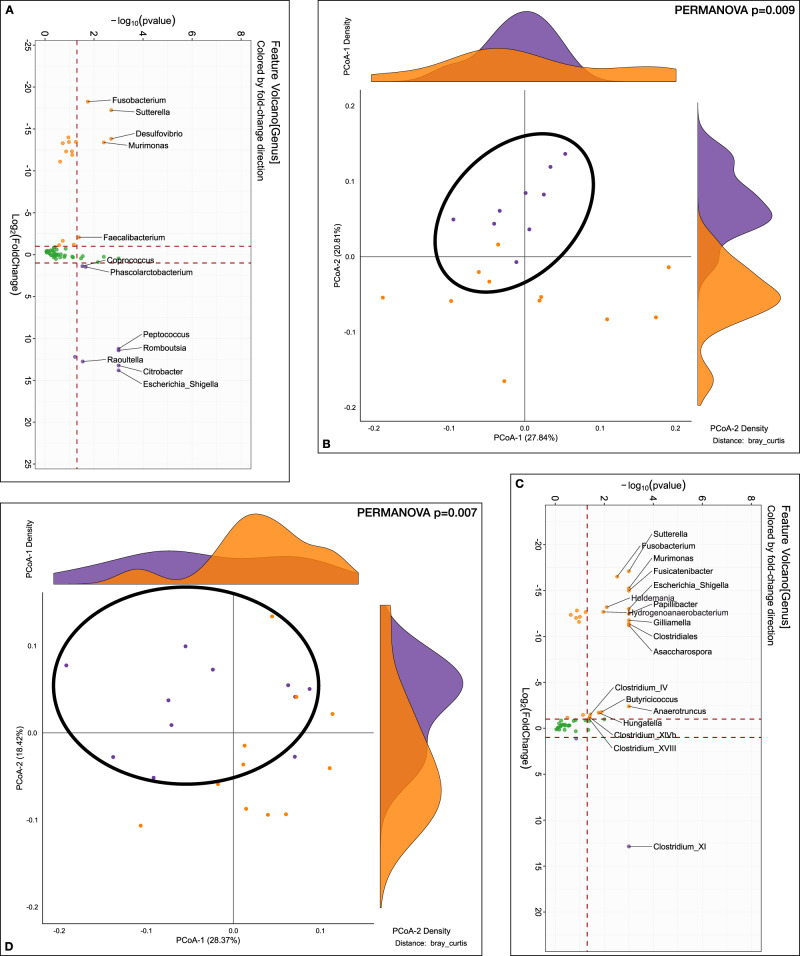
Stool microbiota analyses before and after alcohol preference experiment. **A**, **B** Comparison between mouse recipients’ fecal samples at genus level before alcohol exposuret, Purple: Pre-FMT, Orange: Post-FMT. A log2fold changes Volcano plotted against the log10 *p* value differences between the pre-FMT (purple) and post-FMT (orange). B β-diversity analysis using Bray-Curtis distance on PCoA showing significant separation between groups (PERMANOVA, *p* = 0.009). **C**, **D** Comparison between mouse recipients’ fecal samples at genus level after alcohol preference experiment, Purple: Pre-FMT, Orange: Post-FMT. **C** log2fold changes Volcano plotted against the log10 *p* value differences showing greater abundance of genera post-FMT (orange). **D** β-diversity analysis using Bray-Curtis distance on PCoA showing significant separation between groups (PERMANOVA, *p* = 0.007).

Before alcohol preference analyses showed that Pre-FMT mice were enriched in Proteobacteria genera such as *Escherichia_Shigella, Raoultella*, *Citrobacter* as well as *Phascolarcobacterium* and *Peptococcus* and had a lower abundance of *Faecalibacterium*, *Fusobacterium, Desulfovibrio, Sutterella* and *Murimonas* versus post-FMT mice (Fig. 5A). There was a significant separation on PERMANOVA (Fig. 5B).

After alcohol drinking, the significant difference in PERMANOVA beta-diversity was again seen (Fig. 5C). These mice also had lower alpha-diversity. Post-alcohol, the post-FMT group showed higher abundance of *Sutterella*, *Fusobacterium, Murimonas*, and several genera that are SCFA producers and members of Lachnospiraceae and Ruminococcaceae (Fig. 5D).

**Fig. 6:**
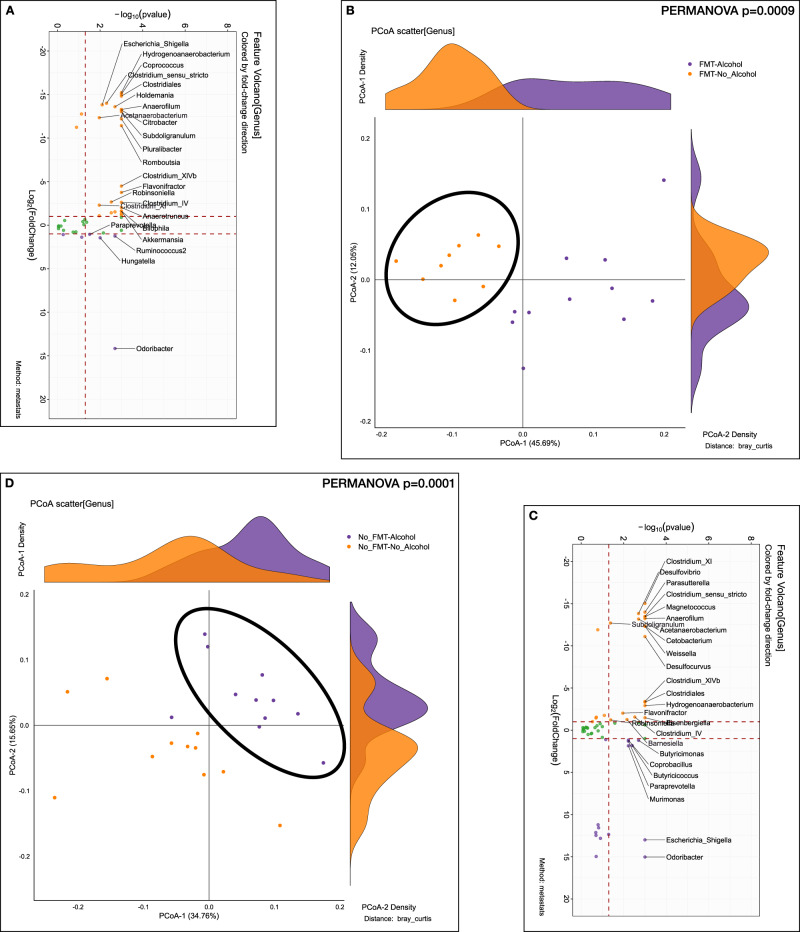
Stool microbiota analyses within the Pre-FMT and within the Post-FMT group before/after alcohol preference. **A**, **B** Comparison between mouse recipients’ fecal samples at the genus level before/after alcohol preference experiment within Pre-FMT. Purple: After alcohol, Orange: Before alcohol. **A** log2fold changes Volcano plotted against the log10 *p* value differences between the before alcohol (orange) and after alcohol (purple). **B** β-diversity analysis using Bray-Curtis distance on PCoA showing significant separation before/after alcohol preference (PERMANOVA, *p* = 0.0009). **C**, **D** Comparison between mouse recipients’ fecal samples at genus level before/after alcohol preference experiment within Post-FMT. Purple: After alcohol, Orange: Before alcohol. **C** log2fold changes Volcano plotted against the log10 *p* value differences between the before alcohol (orange) and after alcohol (purple). **D** β-diversity analysis using Bray-Curtis distance on PCoA showing significant separation before/after alcohol preference (PERMANOVA, *p* = 0.0001).

Within the pre-FMT group, after alcohol intake, there was a significant reduction in the abundance of genera belonging to Lachnospiraceae (*Coprococcus)* and Ruminococcaceae (*Subdoligranulum, Anaerofilum, Anaerobacterium, Anaerotruncus)* as well as Proteobacteria (Fig. 6A orange dots). There was, however, still a higher *Odoribacter*, *Hungatella,* and *Ruminococcus* increase post-drinking compared to baseline. Post-alcohol group of mice had greater separation on PERMANOVA compared to pre-alcohol (Fig. 6B).

Within the post-FMT group, after 48 h of alcohol access, there was a reduction in several Lachnospiraceae and Ruminococcaceae genera as well as sulfur-reducing bacteria (Desulfovibrio, Desulfocurvus), while an increase in other Proteobacterial spp and other species belonging to *Odoribacter, Coprobacillus, Butyricicoccus*, and *Murimonas* were identified (Fig. 6C). PERMANOVA again showed a major separation before/after alcohol drinking (Fig. 6D).

### Specific microbial genera were linked with alcohol intake and preference

**Fig. 7:**
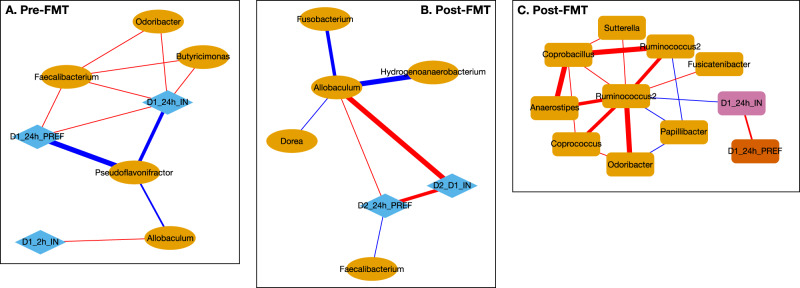
Correlation networks between alcohol intake and preference and stool microbial genera. Red Edge: positive, Blue Edge: negative correlation, D1_2hI_IN: binge intake day 1, D1_24h_PREF: 24 h preference day 1, D1_24h_IN: day 1 intake, D2_24h_PREF: 24 h preference day 2, D2_D1_IN: total intake, Pink nodes are microbial genera. Pre-FMT are mice colonized with stool from donors before FMT, while post-FMT are the separate group of mice colonized with stool from donors after healthy human FMT. **A** Pre-FMT stool colonized mice correlation network with *Faecalibacterium* and Day 1 preference and intake. **B** Post- FMT stool colonized mice network with *Faecalibacterium* and *Allobaculum* for Day 2 intake and preference. **C** Post-FMT network with *Ruminococcus* Day 1 preference and intake.

In the Pre-FMT colonized mice, correlation networks at the genus level showed a positive linkage of *Faecalibacterium*, *Odoribacter* and *Butyricimonas* with day 1 alcohol preference and intake and negative linkage with *Pseudoflavonifactor* (Fig. 7A). *Allobaculum* was positively linked with 2-h intake and negatively with *Pseudoflavonifactor*.

In Post-FMT colonized mice, *Allobaculum* was positively linked with day 2 intake and preference, while *Faecalibacterium* was negatively linked with these activities. *Ruminococcus* spp were negatively linked with day 1 intake and preference and with *Papillibacter* and positively associated with other potential SCFA-producing taxa and with *Sutterella* (Fig. 7B, C).

**Fig. 8:**
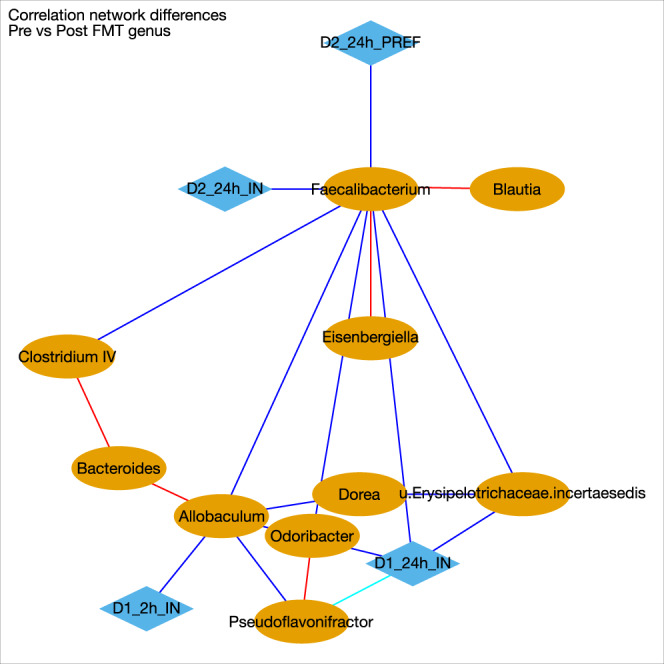
Correlation Differences in mice after colonization. Blue: shift from positive to negative (Pre-FMT to post-FMT), Red: shift negative to positive(Pre-FMT to post-FMT), Cyan: loss in negative correlation from Pre-FMT to post-FMT, which means post-FMT the negative correlation between the 2 nodes that was present pre-FMT is now not present. Pink nodes are microbial genera. D1_2hI_IN: binge intake day 1, D1_24h_IN: 24 hour preference day 1, D2_24h_IN: day 2 intake, D2_24h_PREF: 24 h preference day 2, Pink nodes are microbial genera.

Correlation network differences showed that *Faecalibacterium* correlation shifted from positive in pre-FMT to negative post-FMT with respect to day 1 and 2 alcohol intake (Fig. 8). A similar profile change was seen with *Odoribacter* and *Allobaculum* genera. *Pseudoflavonifactor* was negatively associated with day 1 alcohol intake, but this reduced in intensity in the post-FMT group.

### Small intestine, rather than liver or prefrontal cortex had higher differentially expressed genes on RNA-Seq in post-FMT colonized mice

**Fig. 9:**
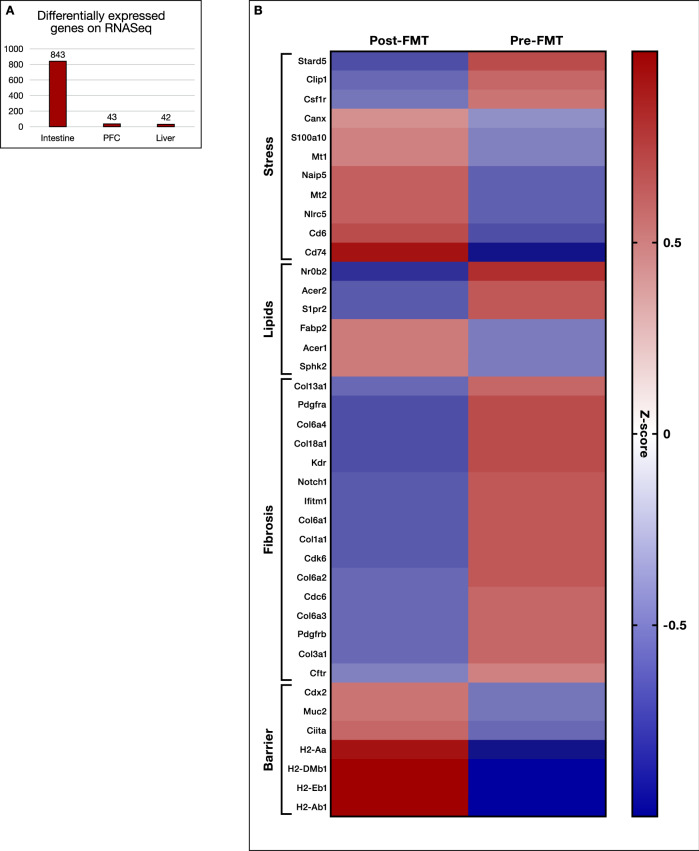
Differentially Expressed Genes (DEGs)on Tissue RNA-Seq. **A** Number of DEGs between pre and post-FMT mice were highest in small intestine compared to liver or prefrontal cortex (PFC). **B** Heatmap of relevant small intestinal genes differentially expressed between pre and post-FMT colonized mice exposed to alcohol arranged according to the functional group.

To understand the signaling changes following colonization and brief ethanol drinking sessions, we performed RNA-Seq analysis on the PFC, liver, and small intestine of 6 pre-FMT and 6 post-FMT mice (GSE205303). As expected, the intestine showed the majority of the differentially expressed genes (DEGs). DESeq2 analysis revealed that 843 genes were differentially expressed at FDR < 0.2 in the intestinal mucosa of pre-FMT versus post-FMT mice (Fig. 9A and Supplemental Data File 1). To further characterize the molecular functions and biological processes of these DEGs, we performed and Gene Ontology and KEGG pathway analysis using DAVID and Revigo to reduce the complexity and redundancy of each of the GO categories using semantic language processing (Supplementary Table 4)^15^. QIAGEN Ingenuity pathway analysis (IPA; QIAGEN Inc., https://digitalinsights.qiagen.com/IPA) was used to identify upstream regulators of these DEGs and canonical pathways overrepresented in this dataset^16^. A number of cellular processes were altered in the small intestine, including immune response, stress response, bile acid signaling, sphingolipid metabolism, epithelial cell renewal, and fibrotic response. As shown in Fig. 9B and Figs. S1 and S2, the key genes related to antigen presentation and antibody production, such as *Cd74*, H-2 class II histocompatibility antigen, A beta 1 chain (*H2-Ab1*), M beta 1 chain (*H2-Dmb1*), A antigen (*H2-Aa*), E-beta 1 chain (*H2-Eb1*) and *Cd6* (the T-cell differentiation antigen), were significantly higher in the post-FMT group compared to the pre-PMT group. Genes related to fibroblast activation and intestine fibrosis, such as collagens, *Pdgfra, Pdgfrb, Cdk6*, and *Cdc6*, were significantly downregulated in the post-FMT group compared to the pre-PMT group. The expression levels of mucin 2 (*Muc2*) and caudal-type homeobox 2 (*Cdx2*), which are involved in intestinal barrier function, were also higher in the post-FMT group compared to the pre-FMT group. *Cdx2* is an intestinal-specific transcription factor and plays a critical role in regulating intestinal barrier function. The expression of calnexin (*Canx*), an important player in regulating endoplasmic reticulum (ER) calcium homeostasis and protein folding, was also upregulated in the post-FMT group. The expression levels of metallothionein (*Mt1* and *Mt2*) were upregulated in the post-FMT group. In addition, the expression levels of small heterodimer partner (*Shp* or *Nrob2*) and sphingosine-1 phosphate receptor 2 (*S1pr2*) were reduced, but the expression of sphingosine kinase 2 (*Sphk2*) was upregulated in the post-FMT group.

In the PFC, 43 DEGs were identified by DESeq2 analysis (Supplemental Data File 1). Gene Ontology analysis revealed only a few over-represented categories that were largely associated with response to estradiol, actin filament network formation, and dendritic spines (Table S4). In the liver, 42 genes were differentially expressed between pre-FMT and post-FMT mice (Supplemental Data File 1). Only three were also in common in the intestine dataset, none were in common in the PFC. DEG changes in the liver were mainly associated with ciliary neurotrophic factor receptor activity, TGF-β activity and cytokine-cytokine receptor interaction (Table S4).

## Discussion

We found that FMT-related decrease in alcohol preference and intake that were found in patients with cirrhosis were transmissible to germ-free mice through intact stool but not using sterile supernatants. Microbial taxa that were higher in post-FMT donors were also associated with lower alcohol intake and preference in the mice.

There is increasing evidence that altered gut-liver-brain axis in AUD can modulate the development of liver dysfunction, cognitive impairment, and immune dysfunction^17,18^. In addition to affecting the liver and brain, AUD has multi-systemic impacts on gut microbiota composition and function in humans^19,20^. These effects synergize to worsen the quality of life of these patients and could potentially be modified using gut microbial manipulation.

In our randomized clinical trial, we found improvement in alcohol craving and consumption at day 15, which then translated into longer-term beneficial AUD-related outcomes^14^. While the focused SCFA improvement was linked to craving, we needed to explore whether alcohol preference and intake changes could also be transmitted to rodent models, get a more expansive view of the effects of FMT and begin to understand the molecular changes occurring in the affected organ systems to alter ethanol intake and preference following FMT.

One of the major findings in this study is that colonization of mice with microbiota from post-FMT AUD patients consumed less than half the amount of ethanol than mice colonized with untreated (baseline) microbiota (Pre-FMT) from the same patients. Initial ethanol acceptance was also reduced in these mice as was ethanol preference. This finding replicates the human impact of the FMT in these patients with cirrhosis, where the craving and consumption of alcohol were reduced from baseline within the FMT group at 15 days^14^. While prior studies have shown changes in neuro-inflammation, socialization, and behavior in mice colonized with stools from patients with differing amounts of alcohol use^12,13^, we extend these by using human samples from a successful trial of alcohol use reduction. Moreover, these effects were not found when sterile supernatants from these same human donors were used. This experience isolates the role of live microbiota without the accompanying cirrhosis or pre-existing cognitive impairments that co-exist in patients with cirrhosis who continue to drink^21,22^. In addition, these extended studies by prior groups to show that alcohol intake can affect the microbial composition and vice-versa and contribute to the growing field of microbial changes in substance abuse disorders^23^.

We only used pooled samples from patients in the FMT group to reduce inter-subject variability since it would be a within-subject comparison. In addition, due to sex differences in susceptibility to alcohol-related injury, only men were included in the human trial and male mice were used in this translational study^24^. Future studies will incorporate females to determine potential sex differences in these effects. Changes in microbiota after FMT in these patients showed higher Ruminococcaceae constituents from the healthy donor. These were then translated into the recipient mice with greater Shannon diversity as well as the relative abundance of taxa belonging to Lachnospiraceae and Ruminococcaceae and lower Enterobacteriaceae constituents such as *Escherichia*_*Shigella*, *Citrobacter*, *Raoultella* and *Pluralibacter*. This was striking because the contrast between microbial composition was maintained across two sets of FMT procedures (healthy donor to human AUD recipient and then from human AUD donors to germ-free mice) with taxa that were enriched the original healthy human donor. Moreover, these same taxa were also differentially associated with alcohol preference and intake at day 1 and day 2 of the experiment. Specifically, *Faecalibacterium, Allobaculum, Sutterella* and selected *Ruminococcus* genera changed relationships with alcohol preference pre versus post-FMT colonization. Partly this could be explained by the higher alcohol intake in the pre-FMT mice but we obtained stool 18 h after the last exposure to alcohol. In patients with alcohol-related liver disease, there is a lower abundance of *Faecalibacterium, Coprococcus*, and higher Enterobacteriaceae, which in the setting of cirrhosis includes lower *Phascolarctobacterium* and other commensals^10,25^. The interplay between Enterobacteriaceae members and *Faecalibacterium* is well known in patients with AUD without cirrhosis^10^. We found the same relationship in the pre-FMT mice that was changed to higher *Faecalibacterium* in post-FMT recipients and was also linked with changes towards lower alcohol intake and preference. These patterns existed even before they were exposed to alcohol, and are consistent with those observed in mice fed alcohol for over 8 weeks, i.e., higher Proteobacteria and lower Firmicutes and obligate anaerobes^11,26^. Moreover, functional changes, including increased intestinal butyrate that would result from higher Ruminococcaceae and Lachnospiraceae members, also showed an increase in mice colonized with post-FMT vs pre-FMT stools. This was accompanied by a lower circulating LBP, which could be indicative of a healthier intestinal barrier after colonization with stools from post-FMT donors compared to pre-FMT^27,28^. This suggests that microbiota from the AUD cirrhosis donors after several decades of alcohol exposure was successfully transmitted into the mice. Acute alcohol exposure in this background further changed microbial taxa correlations, including *Allobaculum* spp changes, as seen in prior studies^11^. In our post-FMT mice that received stools from alcohol-related cirrhosis patients who had received a healthy donor FMT, the baseline is further skewed, which makes these consistent microbial alterations even more striking. Therefore, the phenotypical changes in microbiota were largely replicated from prior human and animal studies and are relatively conserved across two FMT procedures (healthy human donor to AUD human recipient, then to mouse recipient). Moreover, we also found higher butyrate in the post-FMT mice that reflected the human experience, indicating a beneficial functional change as well.

Considering that mice were colonized with different pools of FMT material and all mice underwent 2-bottle choice alcohol drinking, we cannot disentangle the effects of drinking or FMT alone in our gene expression analysis. Few but interesting gene expression and pathway changes occurred in the PFC that suggest FMT treatment and brief ethanol drinking differentially altered synaptic signaling. PFC DEGs were over-represented in categories for long-term synaptic potentiation, actin filament network formation and dendritic spines along with estrogen signaling. Estrogenic metabolites are associated with feminization due to alcohol use and cirrhosis in men^29^. However, these changes were likely dampened by the fact that the mice only had 48 h of alcohol access as compared to those seen in mouse fed alcohol chronically^30^. The few genes altered in the liver were involved in cytokine receptor activation, TGF-β, and IL-6 signaling, suggesting an inflammatory response, which is involved in alcohol-related liver injury and was attenuated after FMT in our study^31^.

A majority of our DEGs were found in the small intestine rather than the liver or PFC, which is striking since alcohol intake and preference changed significantly in the post-FMT mice. Our bioinformatics analysis revealed that the 843 DEGs were primarily involved in regulating the barrier function, immune response, oxidative stress response, inflammation, bile acid, and sphingolipid signaling, and intestinal cell proliferation and fibrosis. Ingenuity Pathway Analysis identified many immune regulators such as *Tgfb1*, *Il10ra*, *Il1b*, *Infg*, and *Tnf* were major upstream regulators of these genes. Sphingolipid signaling was also a major pathway overrepresented in the Gene Ontology and IPA analyses. On KEGG analysis, several key genes pointing towards antigen processing, antibody production and innate immune response were upregulated in the post-FMT group. The higher expression levels of *Cdx2* and *Muc2* in the post-FMT group indicated that the gut microbiome plays a critical role in regulating intestinal mucosal homeostasis. Very strikingly, several fibrotic genes were markedly suppressed in the post-FMT group along with NOTCH1. It has been reported that NOTCH1 contributes to intestinal inflammation by co-operation with TNFα^32^. In addition to the upregulated anti-oxidative stress genes (*Mt1* and *Mt2*) and ER stress modulator (*Canx*), colony-stimulating factor 1 receptor (*Csf1R*) was significantly downregulated in the post-FMT group. Alterations in gene expression in the intestinal mucosa, and to a lesser extent in the liver or PFC, on RNA-Seq were centered on processes that mediate the altered gut-liver axis in alcohol-related liver diseases, including barrier function that was also corroborated by lower serum LBP and higher intestinal SCFAs in post vs pre-FMT mice. S1P-mediated signaling is associated with immune cell infiltration and activation of inflammation in alcohol-related liver injury^33,34^. It has been reported that gut bacteria-derived sphingolipids impact host metabolic pathways^35^. These results suggest that strengthening the intestinal barrier as a result of the more favorable post-FMT microbiome could mediate gut-modulating alcohol intake that is transmissible from humans.

Interpretation of some results of this study is limited by all mice consuming measurable amounts of alcohol, so some of the RNA-Seq changes could represent differences in alcohol intake. However, mouse tissues were harvested 18 h after the last alcohol intake when ethanol is likely no longer present. Mice used here did not have pre-existing liver disease or cirrhosis and therefore did not completely conform to the human donors, which is a limitation. However, despite the absence of cirrhosis or pre-existing liver injury before colonization, the mouse drinking preference recapitulated the findings of the human trial. We also did not perform RNA-Seq in the GF and supernatant-receiving mice. However, the results suggest that neither alcohol-drinking preference or intake, nor change in SCFA, or LBP were noted between the three groups. We also found that several genera of Ruminococcaceae and Lachnospiraceae were found in higher relative abundance post-FMT in the recipient mice in patterns that followed the human donors. These findings indicate that live bacterial communities that produce SCFA and potentially enhance the intestinal barrier could improve alcohol intake and preference in mice. We pooled all human stools pre- and then post-FMT rather than using individual stools for transplantation. Since 80% of patients showed a lowering in craving and consumption 15-days post-FMT, separately inoculating donor materials from individual subjects would have added complexity without the addition of meaningful results beyond the pooled material. In addition, we only performed RNA-Seq on a subset of the mice, but the selection of those with the widest range of drinking intake could have biased our results. Also, this short-term model did not allow us to study liver injury that often accompanies long-term alcohol feeding.

We conclude that fecal transplant-related reduction in alcohol craving and intake can be transmitted to germ-free mice through multiple impacts on microbiota and the intestinal gene transcriptional interface that focus on the intestinal barrier. These changes are associated with microbiota belonging to Ruminococcaceae enriched in the original healthy human donor linked differentially with alcohol use that beneficially changed after human FMT. Differentially expressed genes in the small intestine related to inflammation, barrier function, S1P signaling, estrogen metabolism, and immune presentation were associated with the changes in alcohol drinking intake and preference. Moreover, germ-free supernatants did not replicate these findings. The majority of altered gene expression in mice that exhibited greatest changes in alcohol intake were in the intestinal mucosa rather than the prefrontal cortex or liver, reflecting the importance of the gut and live microbiota as the interface of the altered gut-liver-brain axis in alcohol use disorder.

## Methods

### Human trial

In a previously published phase 1, double-blind, randomized clinical trial (www.clinicaltrials.gov NCT03416751), men with AUD-related cirrhosis with problem drinking (AUDIT-10 > 8)^36^ were randomized 1:1 into receiving one placebo or FMT enema from a donor enriched in Lachnospiraceae and Ruminococcaceae. 6-month safety was the primary outcome. Patients were enrolled from Richmond VA Medical Center and VCU Medical Center Liver Disease clinics after written informed consent. Alcohol craving questionnaire, alcohol consumption (urinary ethylglucuronide/creatinine, Etg), quality of life (QOL), cognition and stool microbiota were tested at baseline and day 15. Patients were seen at 30 days and a 6-month follow-up when serious adverse events (SAE) analysis was performed. The use of the samples in the current study was covered by the original informed consent and IRB approval at the Richmond Veterans Hospital. Patients were compensated for their time and effort which was approved by the IRB as well. The primary outcome was safety at six months. A 6-month follow-up with serious adverse event (SAE) analysis was performed. We included twenty men with AUD-related cirrhosis (65 ± 6.4 yearsModel for End-Stage Liver Disease 8.9 ± 2.7) with similar demographics, cirrhosis, and AUD severity between those assigned to placebo or FMT. The overall results showed the safety and tolerability of the FMT without any infections or SAEs related to it. There was a significant reduction in proportion of FMT-assigned patients who reduced their craving (90% of FMT versus 30% in placebo at day 15, *P* = 0.02). This was accompanied by a lower urinary Etg/creatinine (*P* = 0.03) and improved cognition and psychosocial quality of life in the FMT group. We also found a significant reduction in serum IL-6 and lipopolysaccharide-binding protein with an increased butyrate and isobutyrate in FMT-assigned patients compared to baseline but not in those assigned to placebo. FMT-assigned patients also showed a higher microbial alpha diversity with higher Ruminococcaceae and other microbiota associated with SCFA production. This was correlated with SCFA levels. On longer-term follow-up at 6 months, we found that placebo group had a higher proportion of patients with any SAEs (80% vs. 20%, *P* = 0.02), AUD-related SAEs (70% vs. 10%, *P* = 0.02), and median SAEs/patient (median [interquartile range], 1.5 [1.25] vs. 0 [0.25] in FMT, *P* = 0.02) compared to those assigned to FMT.

### Stool preparation and sterile supernatant to colonize GF mice

Fresh stools from the subjects (baseline and day 15 post-FMT) were collected and individually mixed with pre-reduced anaerobic solution (0.5% Yeast Extract and 0.05% L-cysteine hydrochloride)^37^. Each patient provided one stool sample so the final pool contained all 10 samples pre and 10 samples post. The samples were centrifuged at 16,000 × *g* for 5 min to remove sediment to prevent clogging of gavage needles. Finally, all samples from each category (pre-FMT, post-FMT) were pooled. We created aliquots of 0.5 g of mixed stool pellets, which were stored at –80 °C. An aliquot of each of the pooled fecal suspensions generated above was further centrifuged at 15,000 × *g* for 20 min to pellet whole cells. The supernatant fluid was filtered and sterilized by passage through a 0.22-micron filter to remove bacteria and other microorganisms. Sterile supernatants were monitored for contamination by inoculating on Brain Heart Infusion culture plates under aerobic and anaerobic conditions and gram-stained for examination under oil immersion microscopy (1000×). Before colonization, these were thawed, resuspended in 1.5 ml of sterile PBS per tube/pellet and then gavaged 0.2 ml per mouse per day (for stool pellets and supernatants). In our prior studies, stool treated in this manner, once thawed and inoculated, could effectively colonize GF mice without complications and with good retention of bacterial profiles^38,39^.

We used pooled donor material to avoid inter-individual variations and to reduce the analytic complexity, resource utilization, and logistic challenges of multiple donor FMT.

### Colonization of GF mice

Using established protocols that the National Gnotobiotic Rodent Resouce Center (NGRRC) has developed and published, intact fecal material or supernatant (depending on group) was transferred to 10–15-week-old GF C57BL/6J male mice by daily orogastric gavage for 3 consecutive days (Fig. 2B)^38–41^. The mice were obtained from the GF breeding isolators of the UNC NGRRC and housed in sterile individually filtered cages with autoclaved food, water and bedding at 72° room temperature and 12 h light/dark cycles for 15 days. Stool was then collected and the mice underwent the alcohol preference experiment. This 2-week duration has proven adequate to ensure satisfactory stable microbial colonization and host adaption within mice in our prior studies^39^. Mice receiving different donor materials were housed in separate cages (1–5 mice/cage) to prevent cross-contamination^42^. As shown in Fig. 1B, one group of mice were not colonized and served as GF controls.

### Drinking experiment

Mice then were singly housed in a standard clean cage on a 12 h light cycle (lights ON 6AM) and offered two bottles of solution. One bottle contained 20% ethanol (v/v) diluted in tap water and the other bottle contained tap water^43–46^. Bottle volume readings were taken 2 h (2 h into the dark cycle), 24 h, and 48 h after bottle placement. Fresh ethanol and water were given and body weights were measured each day^47,48^. Ethanol intake (g/kg body weight), ethanol preference (calculated as (mLs of ethanol)/(mLs of ethanol + mLs of tap water)) and total fluid intake (calculated as mLs of ethanol + mLs of tap water) were calculated and compared between groups using non-parametric tests (Mann–Whitney) as well as two way repeated measures ANOVA analyses.

Bottles were removed on day 3. Sacrifice was performed 18 h after the alcohol bottles were removed. At sacrifice, stool, small intestinal mucosa, prefrontal cortex (PFC), and liver were harvested.

Stool was analyzed for microbiota composition for pre and post-alcohol preference from both groups as noted below. We performed RNA-Seq for six highest drinkers in the pre-FMT and six lowest alcohol drinkers in the post-FMT group on liver, intestinal, and PFC tissues. Experimenters were blinded to the treatment groups.

### Microbiota analysis

Microbial DNA was isolated from stool samples as previously described from samples collected from patients during the trial and mice before and after the FMT^14,49^. Samples were kept at −80 °C until the time of analysis. Bacterial 16S ribosomal RNA (rRNA) gene sequencing: The V1 and V2 variable regions of the bacterial 16SrRNA gene were sequenced using Multitag fusion primers and sequenced on an Ion Torrent PGM next-generation sequencer (supplemental methods). Data from each pooled sample were “deconvoluted” by sorting the sequences into bins based on the barcodes using custom PERL scripts. Thus, we were able to normalize each sample by the total number of reads from each barcode. We utilized a local installation of the RDP11 Classifier (RDP11.5) to produce the taxonomic relative abundance tables used by BiomMiner for comparing Bray-curtis distance using PERMANOVA for β-diversity and DESeq2 for individual taxon analyses^50,51^. We performed the following analyses: pre-FMT vs. post-FMT stools at baseline, pre-FMT before and after alcohol drinking, and post-FMT before and after alcohol drinking^52^. Correlation networks with alcohol intake and preference and bacteria at the genus level were performed in pre-FMT mice, then post-FMT mice, and finally using a correlation network difference analysis. Correlation network differences focused on linkages between the same nodes that changed in the post-FMT network compared to the pre-FMT network. All analyses were performed using published techniques in R^53,54^.

### SCFA analysis

small intestinal contents from all five mouse groups were analyzed using published LC-MS/MS techniques for major SCFAs (acetate, propionate, butyrate, isobutyrate and isocaproate)^55^. LC–MS/MS analysis was carried out using a Shimadzu CBM-20A CL communications bus module, equipped with two LC-30 AD CL pumps, a DGU-20A3R and a DGU-20A5A degassing unit, a SIL-30AC CL autosampler and a CTO-20A CL column-oven. The MS analysis was done using an LCMS-8060 CL triple quadrupole instrument with an electrospray negative ionization source (Shimadzu, Japan). Data acquisition and analysis were performed by Labsolutions insight. Chromatographic separation was carried out on a Thermo Scientific Hypersil Gold C18 column (100 mm × 2.1 mm, 1.9 µm). The flow rate of mobile phase was 0.25 mL/min, accompanied with an injection volume of 1 µL. Mobile phase A was 0.1% formic acid in water, while mobile phase B was acetonitrile. The gradient was optimized at 5% to 50% B in 16 min, maintained 50% B for 2 min, and then hold 80% B for 1 min. The column was equilibrated with 5% B for 2 min.

The LBP levels in the serum were measured by ELISA using the Mouse LBP SimpleStep ELISA Kit from Abcam (Cat# ab269542). Total RNA was isolated from small intestinal tissues using Trizol reagent (QIAGEN) and reverse-transcribed into first-strand cDNA using a High-Capacity cDNA Reverse Transcription Kit (Applied Biosystems).

### RNA-Seq processing and differential expression analysis

FastQC (version 0.11.9) was used to measure QC metrics for all RNA-Seq fastq.gz samples. RNA-Seq data were then aligned to the mouse genome (GRCm39) using STAR (version 2.7.9a) and counts were generated with HTSeq (version 0.13.5). The MultiQC (version 1.11) suite was then used to generate and collate QC metrics for fastq.gz, alignment, and raw count samples. Genes with very low counts across the study (defined as fewer than 10 counts in more than 2 samples) were eliminated before differential expression analysis. Low count genes were determined separately for each tissue type. The DESeq2 package (release 3.13) for R was then used to measure differential expression between pre-FMT and post-FMT mice in the intestine, liver, and PFC. Benjamini and Hochberg False Discovery Rate (FDR) was used to correct for multiple testing with FDR ≤ 0.2 considered significant.

### Biological enrichment

Genes with significant differential expression between pre-FMT and post-FMT mice were further explored for biological enrichment using DAVID Functional Enrichment Chart (v2021q4), with a particular interest in enrichment for Gene Ontology (GO) Categories and KEGG Pathways. QIAGAN Ingenuity Pathway Analysis (IPA; QIAGEN Inc., https://digitalinsights.qiagen.com/IPA) assesses the biological significance and relationships between these genes, based on current scientific literature^16^. Genes with enrichment *p*-values (corrected for multiple testing) at FDR ≤ 0.2 were considered significant. Approvals were obtained from institutional review boards (IRBs) at Richmond VA Medical Center (Protocol BAJAJ0021) and from Institutional Animal Care and Use Committees (IACUCs) at the University of North Carolina Chapel Hill (Protocol 18-266.0-C) and VCU (Protocols AD10001212 and AD20168) before study initiation. All animals received humane care according to the criteria outlined in the *Guide for the Care and Use of Laboratory Animals*.

### Reporting summary

Further information on research design is available in the Nature Research Reporting Summary linked to this article.

## Supplementary information

Supplementary Information

Description of Additional Supplementary Files

Supplementary Data 1

Reporting Summary

## Data Availability

The RNA-Seq data generated in this study have been deposited in the Gene Expression Omnibus (GEO) system https://www.ncbi.nlm.nih.gov/geo/query/acc.cgi?acc=GSE205303), while the microbial data is at with the accession code PRJNA887441. Individual human data cannot be shared due to restrictions by the IRB; composite human data are shown in the manuscript. All other data generated or analyzed during this study are included in this published article (and its supplementary information files) or can be obtained from the corresponding author on reasonable request. Source data are provided with this paper.

## Source data

Source Data
