# Supplementary material for: Reduced alcohol preference and intake after fecal transplant in patients with alcohol use disorder is transmissible to germ-free mice
Source: Nat Commun. 2022 Oct 19;13:6198. doi: 10.1038/s41467-022-34054-6 (PMC9581985; doi:10.1038/s41467-022-34054-6)
Supplement: Supplementary file 1 — Supplementary Information [file 41467_2022_34054_MOESM1_ESM.pdf]

Supplementary Material

1. Methods
2. Supplementary figure 1
3. Supplementary figure 2
4. Supplementary table 1
5. Supplementary table 2
6. Supplementary table 3
7. Supplementary table 4
8. Supplementary data file

Supplementary Methods:

DNA Extraction, PCR, and Multitag Sequencing

DNA was extracted from samples using FastDNA spin kit for Soil (MP Biomedicals) according to manufacturer's protocol with slight modifications.. The DNA was diluted with ultra pure water (1:5-1:10 ratio) to use in PCRs.

Approximately 10 ng of DNA was used in a 20 μl reaction for PCR amplification. AmpliTaq Gold™ DNA Polymerase (Thermo Fisher Scientific, Waltham, Massachusetts, USA) was used for the amplification. Master mix for the 16S rRNA gene amplification was subjected to ultraviolet (UV) light (Stratagene Stratalinker®) prior to the addition of primers and dNTPs to eliminate possible bacterial DNA contamination. UV exposure was titrated to ensure no impact on enzyme activity. Universal 16S rRNA bacterial primers 27F and 357R (Lane, 1991) targeting variable regions 1 and 2, were used for bacterial identification. A standard reaction was performed using a GeneAmp 9700 thermocycler (Applied Biosystems Inc.) with 11 minutes of initial denaturation at 95°C, followed by 32 cycles of 30sec at 95°C, 30sec at 48°C, and 2 minutes at 72°C with added 5sec/cycle, and one cycle of 30 minutes at 72°C and it was hold at 4°C. The longer initial denaturation time is recommended by manufacturer to use with Taq Gold polymerase. This enzyme is attached to an antibody and needs to be activated at 95°C with a

longer incubation time. This helps with longer reaction preparations and to avoid producing non-specific products. Most polymerases as well as Taq Gold add an A base to the end of PCR product and the longer (30 minutes) extension time ensures adding an A to all amplified fragments to avoid the size difference when examining the product length in fingerprints which might be only one base different.

Escherichia coli (E.coli) DNA was used as a positive PCR control and no DNA was used as a negative control for all PCRs. Duplicate LH-PCRs (Length Heterogeneity PCR) were completed and fingerprinted prior to sequencing in order to select the most consistent products to represent the samples for sequencing (Sikaroodi and Gillevet 2012). This quality control step used fusion primers for the bacterial 16S rRNA gene (27F and 357R). Fusion primers contain an adapter joined to an 8 base “barcode”, as well as the appropriate primers. The reverse primer (357R) was FAM labelled on the 5-prime end. The fingerprint was run on an ABI 3130*xl* Fluorescent Sequencer (Applied BioSystems). PCR products were selected based on the fingerprints and pooled. The pool was purified twice (to ensure elimination of primer dimers and short products) with Agencourt AMPure solution (Beckman Coulter) in preparation for sequencing. The purified product was quantified using a DTX880 Multimode Fluorescent detector (Beckman Coulter) and the correct concentration was calculated to use in emulsion PCR prior to sequencing. We used Ion Torrent technology (Thermo Fisher Scientific) with the Personal Genome Machine (PGM) for high-throughput sequencing. All emulsion PCR and sequencing steps were executed using the kits and manufacturer’s protocols for the PGM. A customized PERL script was used to “demultiplex” raw sequence data from each pooled sample and to separate the sequences into individual samples based on the barcodes used for each sample at initial PCR.

Figure S1 legend:

Sphingosine 1 phosphate signaling pathway was overrepresented as a canonical pathway activated in Pre-FMT versus post-FMT mice. Pink indicates higher expression in pre-FMT mice, Green indicates higher expression in post-FMT mice. FMT differentially expressed genes (DEG) using ingenuity pathway analysis (IPA).

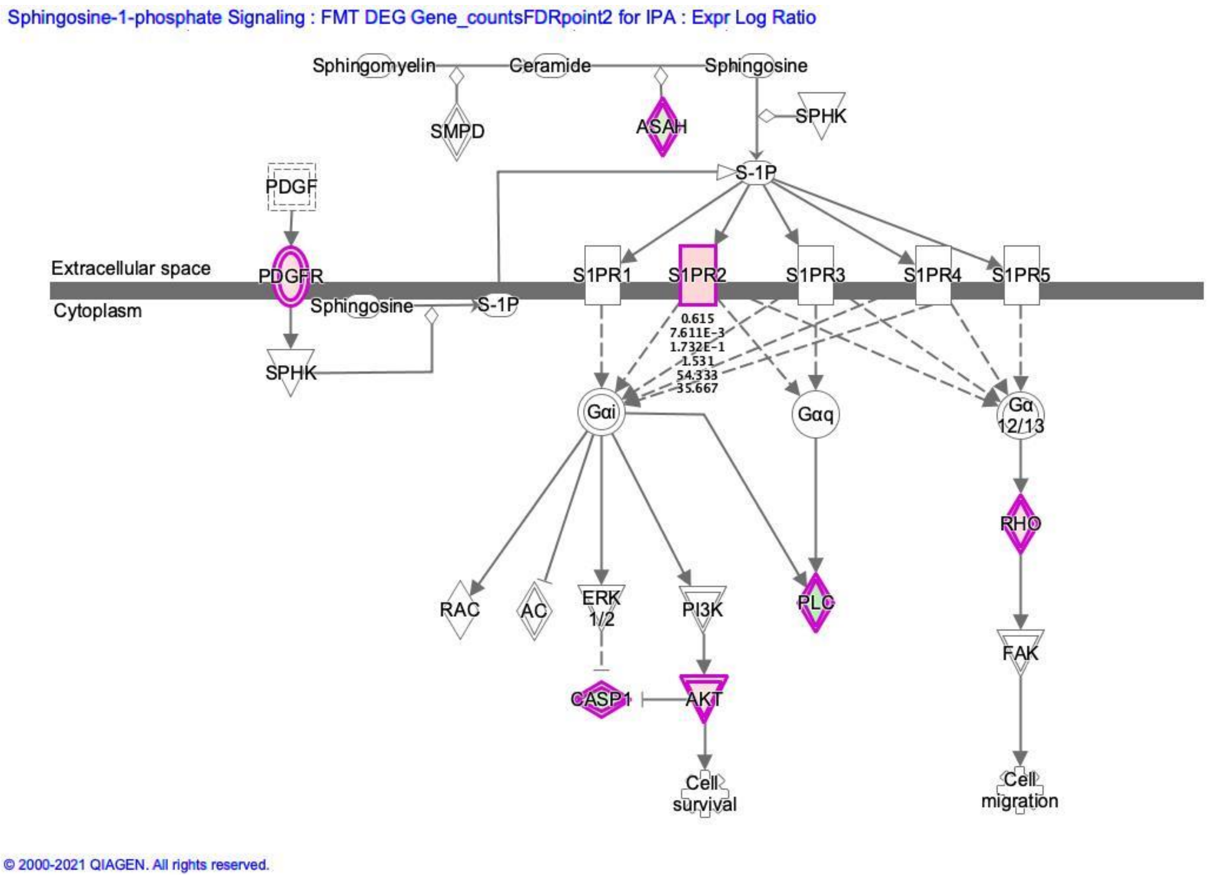

Figure S2 legend: Antigen presentation pathway IPA analysis Pink indicates higher expression in pre-FMT mice, Green indicates higher expression in post-FMT mice. FMT differentially expressed genes (DEG) using ingenuity pathway analysis (IPA).

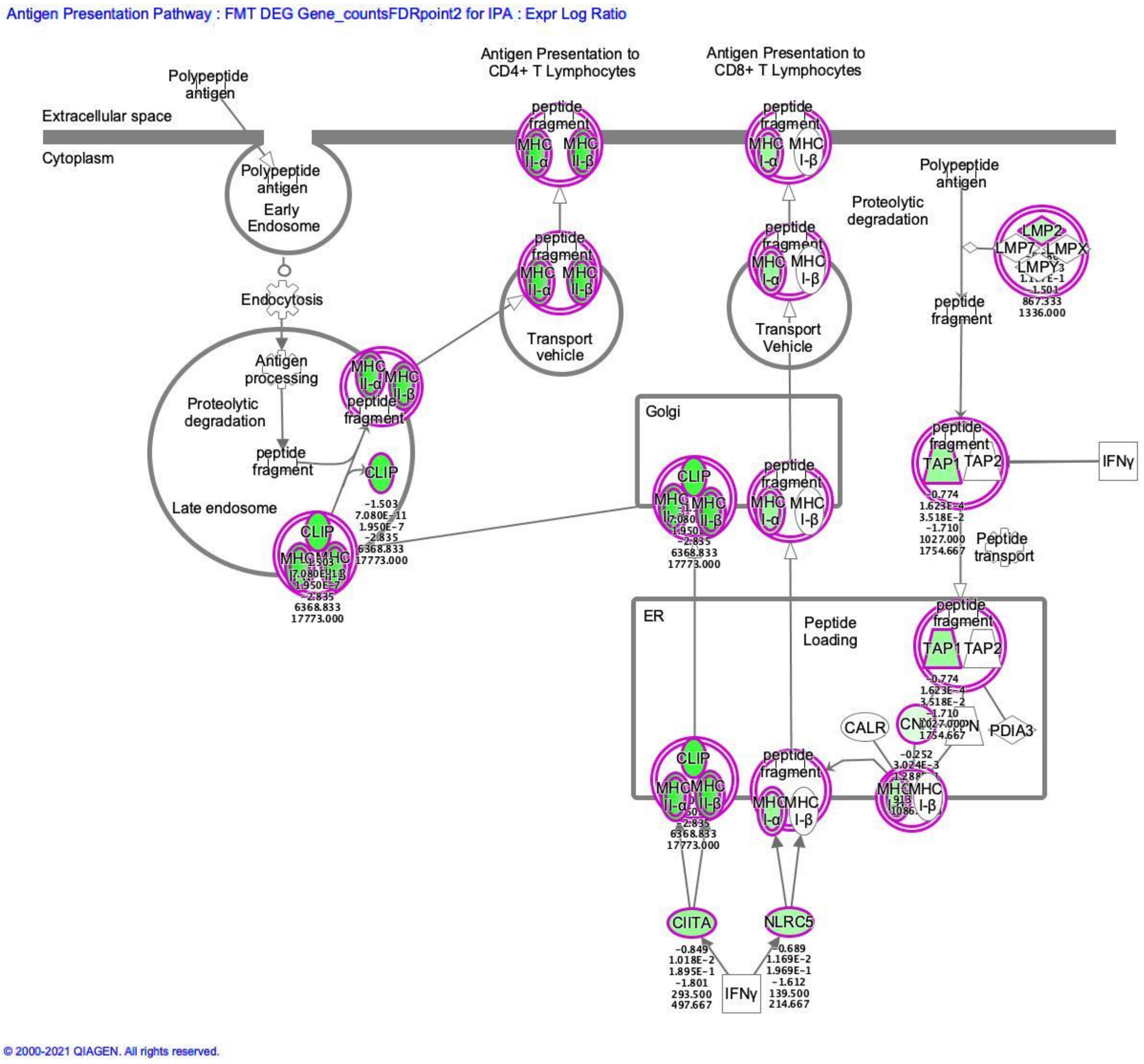

Table S1: Change in Clinical, Patient-reported and Cognitive Outcomes in the human trial

	Placebo (n=10)		FMT (n=10)	
	Pre (baseline)	Post (day 15)	Pre	Post (day 15)
MELD score (range)	9.5±2.8 (8.0)	8.3±2.6 (8.0)	9.3±2.6 (7.0)	8.6±2.8 (7.0)
White blood cell count ($X10^3$/ml range)	6.5±1.3 (3.3)	7.4±1.1 (4.2)	6.2±1.5 (3.1)	6.7±1.4 (4.1)
Aspartate aminotransferase (IU/L range)	40.2±16.7 (53.0)	43.8±29.5 (56.0)	40.5±17.1 (59.0)	58.6±44.3 (118.0)
Alanine aminotransferase (IU/L range)	33.5±22.2 (60.0)	31.9±10.3 (60.0)	34.5±18.9 (32.0)	35.5±10.3 (30.0)
Alkaline phosphatase (IU/L range)	106.2±30.8 (101.0)	104.5±28.2 (83.0)	119.8±21.4 (106.0)	116.0±47.7 (161.0)
Serum albumin (g/dl range)	3.8±0.4 (1.2)	3.7±0.3 (0.9)	3.8±0.33 (1.1)	3.9±0.4 (1.2)
Urinary Etg/creatinine (µg/g creatinine Median (IQR)	0.14 (0.02-0.23)	0.13 (0.02-0.48)	0.12 (0.01-0.86)	0.02 (0.00-0.16)†
**Patient-Reported Outcomes**				
ACQ-SF (high=worse) Median (IQR)	2.7 (1.8-4.7)	3.0 (2.2-5.4)	3.1 (2.4-4.5)	2.5 (2.1-4.3)*
Total SIP (high=worse, range)	12.4±11.7 (39.1)	10.9±11.1 (37.7)	12.3±8.8 (29.6)	9.0±9.1* (18.4)
Physical SIP (high=worse, range)	11.2±9.7 (28.4)	8.6±9.6 (28.6)	8.7±8.0 (24.1)	8.2±7.9 (20.6)
Psychosocial SIP (high= worse, range)	11.3±14.4 (48.3)	10.0±14.2 (48.3)	12.2±13.0 (40.9)	7.0±7.8‡ (21.7)
**Cognitive Testing** Median (IQR)				
PHES (high=better performance)	-6.0(-13.0- -3.5)	-5.5 (-13.0- -1.75)	-5.5(-10.00- 0.0)	-2.5 (-9.25- 1.00)†
EncephalApp(high=poor performance)				
OffTime (seconds)	92.1(86.4-104.9)	93.7 (78.0-104.7)	82.9 (76.0-97.9)	85.3 (75.1-99.7)
OnTime (seconds)	108.3 (98.1-126.5)	104.3 (89.1-149.8)	111.5 (91.1-124.8)	101.7 (93.3-110.9)†
OffTime+OnTime (seconds)	201.3 (184.9-230.3)	185.6 (173.9-237.2)	197.8 (164.7-222.1)	187.5 (167.8-213.3)

Data are presented as mean±SD unless mentioned otherwise. Comparison using one-sided Wilcoxon Signed Rank Paired test within groups, Etg: Ethylglucuronide, ACQ-SF: alcohol craving questionnaire-short form, SIP: sickness impact profile, PHES: Psychometric hepatic encephalopathy score, MELD: model for end-stage liver disease (a high score indicates poor outcome) *p=0.02, ‡p=0.01, †p=0.05

Table S2: Total liquid consumed by C57BL/6 mice during drinking sessions

**Liquid consumed (ml)**	**Binge (2hr)**	**Day 1 (24 hr)**	**Day 2 (24 hr)**
Pre-FMT stool	0.400 (0.225)	3.667 (1.100)	4.233 (2.125)
Post-FMT stool	0.600 (0.225)	4.117 (0.400)	4.533 (1.450)
Germ Free	0.483 (0.650)	4.200 (1.750)	5.175 (1.350)
Pre-FMT supernatant	0.433 (0.089)	4.600 (3.550)	4.175 (2.475)
Post-FMT supernatant	0.333 (0.200)	4.600 (0.925)	3.975 (2.150)

Data presented as median (IQR); No significant changes between and within groups using Mann-Whitney, Kruskal-Wallis and RMANOVA tests.

Table S3: Body weights of FMT mice during the drinking experiment

**Body weight (gm)**	**Day 1**	**Day 2**
Pre-FMT stool	23.75 (1.50)	23.95 (1.68)
Post-FMT stool	23.10 (1.78)	23.60 (1.65)
Germ-free	26.10 (1.65)	25.15 (3.88)
Pre-FMT supernatant	24.35 (2.20)	22.40 (1.80)
Post-FMT supernatant	25.55 (4.53)	24.25 (4.35)

Data presented as median (IQR); No significant changes between and within groups on Wilcoxon paired tests for within and Mann Whitney between groups

Table S4: Gene Ontology and KEGG pathway over-representation analysis of differentially regulated genes in the intestine, prefrontal cortex and liver of pre-FMT vs. post-FMT mice.

Intestine				
**KEGG Pathway**	**Description**	**Overlap**	**Category Total**	**p-value**
mmu04510	Focal adhesion	25	314	3.2e-06
mmu04512	ECM-receptor interaction	15	314	1.1e-05
mmu04151	PI3K-Akt signaling pathway	30	314	2.0e-04
mmu04014	Ras signaling pathways	22	314	4.0e-04
mmu05145	Toxoplasmosis	13	314	0.0011
mmu04612	Antigen processing and presentation	11	314	0.0017
mmu05231	Choline metabolism in cancer	12	314	0.0026
mmu05323	Rheumatoid arthritis	10	314	0.0059
mmu04514	Cell adhesion molecules (CAMs)	15	314	0.0062
mmu04020	Calcium signaling pathway	16	314	0.0065
mmu04672	Intestinal immune network for IgA production	7	314	0.0066
mmu04015	Rap1 signaling pathway	17	314	0.014
mmu04024	cAMP signaling pathway	16	314	0.014
mmu05310	Asthma	5	314	0.015
mmu05150	Staphylococcus aureus infection	7	314	0.015
mmu04915	Estrogen signaling pathway	10	314	0.018
mmu00480	Glutathione metabolism	7	314	0.023
mmu04974	Protein digestion and absorption	9	314	0.026
mmu04142	Lysosome	11	314	0.027
mmu05152	Tuberculosis	14	314	0.027
mmu04610	Complement and coagulation cascades	8	314	0.034
mmu04940	Type I diabetes mellitus	7	314	0.039
mmu04611	Platelet activation	11	314	0.041
mmu4724	Glutamatergic synapse	10	314	0.044
mmu01100	Metabolic pathways	64	314	0.048
mmu04010	MAPK signaling pathway	17	314	0.050
**Prefrontal Cortex**				
**GO category**	**Description**	**Overlap**	**Category Total**	**p-value**
GO:0007507	Heart development	4	34	0.012
GO:0032355	Response to estradiol	3	34	0.015
GO:0030336	Negative regulation of cell migration	3	34	0.016

GO:0051639	Actin filament network formation	2	34	0.020
GO:0051764	Actin crosslink formation	2	34	0.022
GO:0007275	Multicellular organism development	6	34	0.037
GO:0042995	Cell projection	7	38	0.0020
GO:0005911	Cell-cell junction	4	38	0.0063
GO:0030054	Cell junction	6	38	0.011
GO:0001726	Ruffle	3	38	0.014
GO:0005615	Extracellular space	8	38	0.020
GO:0043197	Dendritic spine	3	38	0.032
GO:0005802	Trans-Golgi network	3	38	0.038
GO:0005737	Cytoplasm	19	38	0.043
GO:0015293	Symporter activity	3	35	0.020
GO:0005515	Protein binding	14	35	0.038
**Liver**				
**GO category**	**Description**	**Overlap**	**Category Total**	**p-value**
GO:0070120	Ciliary neurotrophic factor-mediated signaling pathway	2	23	0.0061
GO:0048589	Developmental growth	2	23	0.042
GO:0004897	Ciliary neurotrophic factor receptor activity	2	26	0.0057
