# Supplementary material for: Reduced alcohol preference and intake after fecal transplant in patients with alcohol use disorder is transmissible to germ-free mice
Source: Nat Commun. 2022 Oct 19;13:6198. doi: 10.1038/s41467-022-34054-6 (PMC9581985; doi:10.1038/s41467-022-34054-6)
Supplement: Supplementary file 2 — Description of Additional Supplementary Files [file 41467_2022_34054_MOESM2_ESM.pdf]

Description of Additional Supplementary Files

File Name: Supplementary Data 1
Description: Differentially expressed genes in the intestine, liver and prefrontal cortex (PFC) between pre and post-FMT groups (excel sheet). Fold change (pre-FMT vs. post-FMT), p-values and adjusted p-values are reported for genes that were significant at a false discovery rate <0.2."
