# Supplementary material for: Reduced alcohol preference and intake after fecal transplant in patients with alcohol use disorder is transmissible to germ-free mice
Source: Nat Commun. 2022 Oct 19;13:6198. doi: 10.1038/s41467-022-34054-6 (PMC9581985; doi:10.1038/s41467-022-34054-6)
Supplement: Supplementary file 4 — Reporting Summary [file 41467_2022_34054_MOESM4_ESM.pdf]

nature portfolio

Corresponding author(s): Jasmohan Bajaj

Last updated by author(s): Sep 21, 2022

Reporting Summary

Nature Portfolio wishes to improve the reproducibility of the work that we publish. This form provides structure for consistency and transparency in reporting. For further information on Nature Portfolio policies, see our Editorial Policies and the Editorial Policy Checklist.

Statistics

For all statistical analyses, confirm that the following items are present in the figure legend, table legend, main text, or Methods section.

n/a	Confirmed	
☐	☒	The exact sample size (*n*) for each experimental group/condition, given as a discrete number and unit of measurement
☐	☒	A statement on whether measurements were taken from distinct samples or whether the same sample was measured repeatedly
☐	☒	The statistical test(s) used AND whether they are one- or two-sided<br>*Only common tests should be described solely by name; describe more complex techniques in the Methods section.*
☐	☒	A description of all covariates tested
☐	☒	A description of any assumptions or corrections, such as tests of normality and adjustment for multiple comparisons
☐	☒	A full description of the statistical parameters including central tendency (e.g. means) or other basic estimates (e.g. regression coefficient) AND variation (e.g. standard deviation) or associated estimates of uncertainty (e.g. confidence intervals)
☐	☒	For null hypothesis testing, the test statistic (e.g. *F*, *t*, *r*) with confidence intervals, effect sizes, degrees of freedom and *P* value noted<br>*Give P values as exact values whenever suitable.*
☒	☐	For Bayesian analysis, information on the choice of priors and Markov chain Monte Carlo settings
☒	☐	For hierarchical and complex designs, identification of the appropriate level for tests and full reporting of outcomes
☒	☐	Estimates of effect sizes (e.g. Cohen's *d*, Pearson's *r*), indicating how they were calculated

Our web collection on statistics for biologists contains articles on many of the points above.

Software and code

Policy information about availability of computer code

Data collection	no specific software for data collection was used.
Data analysis	DESeq2 (release 3.13), STAR (version 2.7.9a), FastQC (version 0.11.9) HTSeq (version 0.13.5), MultiQC (version 1.11) suite, DAVID functional enrichment chart (v2021q4 https://david.ncifcrf.gov/), Gene ontology (GO, http://geneontology.org/), KEGG (www.kegg.jp), Ingenuity Pathway Analysis (www.ingenuity.com), Graphpad PRISM for data analysis

For manuscripts utilizing custom algorithms or software that are central to the research but not yet described in published literature, software must be made available to editors and reviewers. We strongly encourage code deposition in a community repository (e.g. GitHub). See the Nature Portfolio guidelines for submitting code & software for further information.

Data

Policy information about availability of data

All manuscripts must include a data availability statement. This statement should provide the following information, where applicable:

- Accession codes, unique identifiers, or web links for publicly available datasets
- A description of any restrictions on data availability
- For clinical datasets or third party data, please ensure that the statement adheres to our policy

Microbial data is at http://mbac.gmu.edu:8080/u/gillevet/h/alcfmtmousedataselected060122 while RNASeq data is deposited to GEO GEO Submission (GSE205303) [NCBI tracking system #23017667]. Clinical metadata for patients is not available due to ethics restrictions. All these links are working.

Human research participants

Policy information about studies involving human research participants and Sex and Gender in Research.

Reporting on sex and gender	All volunteers in the human trial were of male sex and gender
Population characteristics	The human trial is already fully published and now details are added
Recruitment	The human trial is already fully published; recruitment details are in the revision
Ethics oversight	The human trial is already fully published; IRB at the Richmond VA Medical Center with protocol BAJAJ0021

Note that full information on the approval of the study protocol must also be provided in the manuscript.

Field-specific reporting

Please select the one below that is the best fit for your research. If you are not sure, read the appropriate sections before making your selection.

☒ Life sciences ☐ Behavioural & social sciences ☐ Ecological, evolutionary & environmental sciences

For a reference copy of the document with all sections, see nature.com/documents/nr-reporting-summary-flat.pdf

Life sciences study design

All studies must disclose on these points even when the disclosure is negative.

Sample size	20 men with alcohol-related cirrhosis who were actively drinking. 10 were randomized to FMT and 10 to placebo enemas. The samples used here from the 10 men who received FMT collected before and 15 days post-FMT. These were then put into 14 mice each pre and post. Sterile supernatants were then introduced into 6 GF mice each and 6 GF mice remained as controls. Sample size calculation for the human trial was based on prior Phase 1 studies in FMT. Sample size calculation regarding individual mice was performed according to prior studies by Dr Sartor's lab (Kim et al) that ensure 5-6 mice per category.
Data exclusions	None were excluded from the human samples; 2 mice who received gavage using post-FMT sterile supernatants developed injury due to gavage and had to be excluded.
Replication	No replication analyses were performed but aliquots of stool pre and post-FMT from all 10 subjects were combined to then introduce to the mice. This was due to the low N of the mice and aliquots and
Randomization	Randomization was done at www.random.org for the human samples. The mice were randomly allocated to receive the specific aliquots and germ-free supernatants by the NGRRC.
Blinding	The outcomes assessor and patients were blinded to the randomization for the original trial. The behavioral outcomes assessor for alcohol use was blinded to the source of the colonization in the mice.

Reporting for specific materials, systems and methods

We require information from authors about some types of materials, experimental systems and methods used in many studies. Here, indicate whether each material, system or method listed is relevant to your study. If you are not sure if a list item applies to your research, read the appropriate section before selecting a response.

Materials & experimental systems

n/a	Involved in the study	
☒	☐	Antibodies
☒	☐	Eukaryotic cell lines
☒	☐	Palaeontology and archaeology
☐	☒	Animals and other organisms
☒	☐	Clinical data
☒	☐	Dual use research of concern

Methods

n/a	Involved in the study	
☒	☐	ChIP-seq
☒	☐	Flow cytometry
☒	☐	MRI-based neuroimaging

Animals and other research organisms

Policy information about studies involving animals; ARRIVE guidelines recommended for reporting animal research, and Sex and Gender in Research

Laboratory animals	10-15-week-old GF C57BL/6 male mice from Jackson laboratory housed under GF conditions and then gavaged using specific human materials.
Wild animals	none
Reporting on sex	Only male mice since the human population that provided the samples was male as well.
Field-collected samples	None
Ethics oversight	IACUC at University of North Carolina Chapel Hill (Protocol 18-266.0-C) and VCU (Protocols AD10001212 and AD20168)

Note that full information on the approval of the study protocol must also be provided in the manuscript.
